# The presence of the *dam* gene is important for the proliferation of *Salmonella* Typhimurium in egg yolk

**DOI:** 10.1016/j.psj.2026.107818

**Published:** 2026-09-22

**Authors:** Aisha Madi, Greeshma Bharathan, Ana Victoria Troncoso, Camila Ortiz Melcon, Karoll Rodriguez Chinchilla, Maddie Childress, Michelle Hayden, Krishna Pada Singha, Amit Morey, Stephen White, Shabarinath Srikumar

**Affiliations:** a Department of Poultry Science, College of Agriculture, Auburn University, Alabama, 36849, USA; b School of Fisheries, Aquaculture and Aquatic Sciences, Auburn University, Auburn, AL, 36849, USA; c Poultry Microbiological Safety and Processing Research Unit, U.S. National Poultry Research Center, Richard B. Russell Agricultural Research Center, USDA-ARS, 950 College Station Rd., Athens, GA, 30605-2702, United States

**Keywords:** Egg yolk, Regulatory proteins, *Salmonella* Typhimurium, *Salmonella* Enteritidis, *dam*, *hilD*

## Abstract

Chicken egg is an important vector of global salmonellosis. However, mechanistic insights into *Salmonella* proliferation in eggs, particularly in the yolk, are lacking. Here, we employed a suite of eight regulatory mutants to identify the distinct stresses *Salmonella* encounters during yolk proliferation. The regulatory mutants of *Salmonella* Typhimurium ST4/74 (ST4/74) were inoculated into pasteurized egg yolk, and their growth kinetics were compared individually with those of the isogenic wild type. Of the eight mutants, the null mutant of the *dam* gene, which encodes DNA adenine methyltransferase, exhibited significantly reduced growth compared to the wild type. The presence of the *dam* gene was further demonstrated to be important for *Salmonella* proliferation in bovine milk but not in chicken meat. Null mutants of the gene encoding the SPI-1 regulatory protein HilD and of oxidative stress response proteins such as KatN, SodA, and MntH grew better than the wild type in yolk. Null mutants of the same genes in *S.* Enteritidis P125109 behaved similarly to those in ST4/74, suggesting a more global role for these genes in the *Salmonella* egg yolk survival. Overall, our findings provide early insights into the mechanisms *Salmonella* uses to survive in egg yolk.

## Introduction

Foodborne illness is a global health concern. In 2019 alone, there were about 9.9 million domestically acquired foodborne illnesses in the United States (Walter et al., 2025). Of this, *Salmonella* was the most important, causing 238 deaths, 12,500 hospitalizations, 1.3 million illnesses, and an estimated economic burden of $17.1 billion (Hoffmann, et al., 2024). Many food matrices contribute to *Salmonella* outbreaks. Consumption of contaminated chicken eggs accounted for about 10% of all *Salmonella* outbreaks in the United States (White, et al., 2022). Within eggs, the yolk is the primary supporter of *Salmonella* proliferation, while the albumen is mostly antimicrobial (Gantois, et al., 2009).

*Salmonella* infection is associated with the consumption of contaminated food or water. The pathogen then invades the intestinal epithelium, triggering gastroenteritis (Haraga, Ohlson and Miller, 2008). In some cases, the infection spreads beyond the intestinal epithelium, resulting in systemic dissemination (Kingsley, et al., 2009). The *Salmonella* genome contains multiple *Salmonella* pathogenicity islands (SPIs) that support bacterial pathogenesis (Sia, et al., 2025). A couple of well-characterized SPIs, SPI-1 and SPI-2, encode specialized Type III Secretion Systems (T3SSs). The T3SS encoded by SPI-1 mediates the invasion of host intestinal epithelial cells. Once in the intracellular environment, *Salmonella* encodes a second T3SS, mediated by SPI-2, that aids pathogen survival within a specialized phagosome-like compartment, the *Salmonella* Containing Vacuole (SCV) (Haraga, Ohlson and Miller, 2008).

The capacity to rapidly adapt to a new environment determines the success of *Salmonella* as a pathogen (Rolfe, et al., 2012). The adaptability is conferred by the ability of the organism to rapidly remodel its transcriptomic profile in response to changes in the bacterial microenvironment (Kroger, et al., 2013). Such a flexible transcriptional program must be carefully controlled by DNA-binding regulatory proteins that act at the level of transcription initiation. Investigations involving these DNA-binding regulatory proteins have yielded deep insights into the stress factors encountered by the pathogen during infection and enabled a deeper understanding of *Salmonella* virulence (Colgan, et al., 2016; Fabrega and Vila, 2013).

Although the dynamics of *Salmonella* proliferation in egg yolk are well characterized, the roles and importance of specific regulatory proteins in the pathogen's adaptation to non-host environments, especially food matrices such as egg yolk, remain unknown. Elucidating the role of such regulatory genes in egg yolk survival will not only enable us to understand the various stress factors the pathogen faces but also help us characterize the molecular mechanisms of adaptation. By analyzing the growth kinetics of eight regulatory mutants in pasteurized liquid egg yolk, we show that the *dam* gene encoding the DNA adenine methylase and *hilD,* regulating SPI-1 expression, are important for *Salmonella* survival in egg yolk. In addition to regulatory proteins, we also show that the pathogen could be exposed to oxidative stress during egg yolk survival.

## Materials and methods

### Bacterial strains and growth conditions

*Salmonella* Typhimurium ST4/74 (ST4/74), originally isolated from a calf with salmonellosis (Rankin and Taylor, 1966) and eight isogenic deletion mutants (Colgan, Kroger, Diard, Hardt, Puente, Sivasankaran, Hokamp and Hinton, 2016) were gifted by the Jay Hinton Laboratory. *Salmonella* Enteritidis P125109 (SEN), originally a clinical isolate (Perez-Sepulveda, et al., 2021; Thomson, et al., 2008) was a gift from the Michael McClelland laboratory. All the SEN mutants were constructed in this study. Whenever needed, the strains were revived from the −80°C glycerol stock and streaked onto Lennox broth (LB) agar plates (Sigma-Aldrich, MO, USA). From the plate, a single colony was picked, inoculated into 5 mL of LB broth, and incubated overnight at 37°C in static conditions. This overnight culture was then centrifuged at 10,000 rpm for 5 minutes and washed twice with 1X phosphate-buffered saline (PBS). The optical density at 600 nm (OD_600_) was then adjusted to 0.001 before inoculation into 50 mL of commercially obtained pasteurized liquid egg yolk (Abbotsford Farms, USA) or bovine milk (local store, Auburn, AL, USA) stored in 50 mL Falcon tubes and incubated at 37°C. Aliquots of 100 µL were obtained every 2 hours until 14 h post-inoculation, then serially diluted and spread-plated on Xylose Lysine Deoxycholate agar (Himedia, India). Colonies were counted using a Scan 1200 colony counter (Interscience, UK), and the growth curve was plotted. For the chicken meat experiment, tenderloins were commercially obtained, cut into 1 g pieces, and UV-sterilized for 20 minutes. Overnight bacterial cultures were washed twice in 1X PBS, diluted to 0.5 Macfarland standard, and 10 µL was spot-inoculated onto UV-sterilized tenderloins. Every two hours, each piece was dispensed in 5 mL of sterile 1X PBS and vortexed to release the bacterial cells. Aliquots of 100 µL were serially diluted and spread plated on Xylose Lysine Deoxycholate agar (Himedia, India), and counted using Scan 1200 colony counter (Interscience, UK).

### Construction of the complementation strain STΔ*dam* pBAD^*dam*-np^

The *dam* gene was cloned on the pBAD plasmid using the SLiCE (Seamless Ligation Cloning Extract) procedure (Zhang, Werling and Edelmann, 2012). Initially, the vector pBAD was amplified, excluding the l-arabinose-inducible P*ara* region, by PCR using outwardly directed plasmid-specific primers, pBAD FP and pBAD RP^np^ (Table 1). Then the *dam* gene, with its putative promoter region (300 bp upstream of the dam CDS start ATG), was amplified from the ST4/74 genome by PCR using primers pBAD-dam FP and pBAD-dam RP (Table 1), which harbor 5′-end homology to the vector pBAD. The amplified PCR products were then treated with the restriction enzyme DpnI (New England Biolabs, Ipswich, MA, USA) and purified using the QIAquick PCR Purification Kit (QIAGEN, Hilden, Germany). The standard SLiCE reaction mix contained the following ingredients: 100 ng linear vector, an appropriate amount of insert DNA leading to a 1:1, 2:1, and 4:1 molar ratio of insert to vector, 1 µl 10X Ligation buffer (New England Biolabs), 1 µl SLiCE extract (Crude lysate of Topo10 *Escherichia coli* pKD46 strain), and ddH_2_O made up to a total volume of 10 µl. The SLiCE reaction was incubated at 37°C for 1 h, and 5 µl of the reaction was electroporated into electrocompetent Topo10 *Escherichia coli* cells, incubated at 37°C for 1 hour, and plated on LB ampicillin plates. The ampicillin-resistant colonies were further grown, and the clones carrying the pBAD*^dam-np^* plasmid were confirmed by PCR using Test pBAD FP^np^ and Test pBAD RP (Table 1). From one successful clone, pBAD*^dam-np^* plasmid was isolated using the QIAprep Spin Miniprep Kit (QIAGEN, Hilden, Germany). The purified plasmids were further electroporated into electrocompetent ST4/74Δ*dam* cells, generating the STΔ*dam* pBAD*^dam-np^* strain.

**Table 1:** Primers used to create the different mutants and complementation strains used in this study.

Primer Name	Sequence	Purpose
pBAD FP	AGATTAAATCAGAACGCAGA	Construction of pBAD-*dam*^np^
pBAD RP^np^	CTCTGAATGGCGGGAGTAT	
pBAD-*dam* FP	CAACTCTCTACTGTTTCTCCATGAAAAAAAATCGCGCTTTTTTG	
pBAD-*dam* RP	TCTGCGTTCTGATTTAATCTTTATTTTCTTGCAGGCGTTGC	
Test pBAD FP^np^	GAGATAACCGTTGGCCTCAA	
Test pBAD RP	GGTCAGGTGGGACCACCG	
SENΔ*hilD* FP	GAACATTAAAATAACATCAACAAAGGGATAATATGGAAAATGTAACCTTTGTGTAGGCTGGAGCTGCTTC	Insertion of kanamycin within *hilD* in SEN
SENΔ*hilD* RP	GATACAAAAAATGTTAATGGTTCGCCATTTTTATGAATGTCGATGGCGTACATATGAATATCCTCCTTA	
SEN Test *hilD* FP	CAATCAGACCATTGCCAACAC	Confirmation of *hilD* deletion in SEN
SEN Test *hilD* RP	TCTCAGAGGGAACGGATGAT	
SENΔ*dam* FP	ACAGCCGGAGAAGGTGTAATTAGTTAGTCAGCATGAAAAAAAATCGCGCTGTGTAGGCTGGAGCTGCTTC	Insertion of kanamycin within *dam* in SEN
SENΔ*dam* RP	CTTCTCCTTGAGAATTATTTTCTTGCAGGCGTTGCGACTCCTGGTTGGTACATATGAATATCCTCCTTA	
SEN Test *dam* FP	GAGTGTCGCTGACAGCTATTT	Confirmation of *dam* deletion in SEN
SEN Test *dam* RP	CCATCAGGTGGACATCAATAGG	
ST4/74Δ*katN* FP	AGCCAAAAAATAAATAATAAGCAGGAGGCAATATGTTTCGACACGTAAAAGTGTAGGCTGGAGCTGCTTC	Insertion of kanamycin within *katN* in ST4/74
ST4/74Δ*katN* RP	TTACTTGTCGGAACTCACTTGTCTTCTTTCGGTTCGCCGCAGCCTAGTTCCATATGAATATCCTCCTTA	
ST4/74 Test *katN* FP	TCCCGTTCGGTGTTGAAAG	Confirmation of *katN* deletion in ST4/74
ST4/74 Test *katN* RP	ACTACACCACCTTCTTCGATAAC	
ST474Δ*sodA* FP	CACTGTGCCGCTCGACAATAATGGAGATGATTATGAGTTATACACTGCCAGTGTAGGCTGGAGCTGCTTC	Insertion of kanamycin within *sodA* in ST4/74
ST474Δ*sodA* RP	ACGTGCAATGCAAATTATTTTTTAGCGGCGAAACGCGCTGCTGCTTCGTCCATATGAATATCCTCCTTA	
ST4/74 Test *sodA* FP	CCAACGTAGCCAGACGTATAAA	Confirmation of *sodA* deletion in ST4/74
ST4/74 Test *sodA* RP	TGTCGCTGATGGCAAAGT	
ST474Δ*mntH* FP	ACATAGCAAAGGCTATGTTTTTGAGGCAAAAGATGACTGACAATCGCGTAGTGTAGGCTGGAGCTGCTTC	Insertion of kanamycin within *mntH* in ST4/74
ST474Δ*mntH* RP	CTATCTTTCTGTCTTTATGACAACCCCATCACCGTTCCAACCAGCAACCACATATGAATATCCTCCTTA	
ST4/74 Test *mntH* FP	GAAGGACGCGGTCCATATTT	Confirmation of *mntH* deletion in ST4/74
ST4/74 Test *mntH* RP	CTCGATCGTGAAGGAGAGAAAG	
SENΔ*sodA* FP	CACTGTGCCGCTCGACAATAATGGAGATGATTATGAGTTATACACTGCCAGTGTAGGCTGGAGCTGCTTC	Insertion of kanamycin within *sodA* in SEN
SENΔ*sodA* RP	TCGTGCAATGCAAATTATTTTTTAGCGGCGAAACGCGCTGCTGCTTCGTCCATATGAATATCCTCCTTA	
SEN Test *sodA* FP	TGGAGAGTAAGGCCAACGTA	Confirmation of *sodA* deletion in SEN
SEN Test *sodA* FP	TCGACAGATTCTCGCCAAAC	
SENΔ*mntH* FP	ACATAGCAAAGGCTATGTTTTTGAGGCAAAAGATGACTGACAATCGCGTAGTGTAGGCTGGAGCTGCTTC	Insertion of kanamycin within *mntH* in SEN
SENΔ*mntH* RP	CTATCTTTCTGTCTTTATGACAATCCCATCACCGTTCCAACCAGCAACCACATATGAATATCCTCCTTA	
SEN Test *mntH* FP	GAAGGACGCGGTCCATATTT	Confirmation of *mntH* deletion in SEN
SEN Test *mntH* RP	CTCGATCGTGAAGGAGAGAAAG	
SEN Test *katN* FP	GCATGTTCCCTTCCGATGAA	Confirmation of *katN* deletion in SEN
SEN Test *katN* RP	ACTACACCACCTTCTTCGATAAC	
474qRT *dam* FPv2	GGTGTGATACGCTGTGAAGT	To validate the complementation of *dam* gene
474qRT *dam* RPv2	GAGTCATACGCAGACAGTATGG	
474qRT *rfaH* FPv2	CGCTTTCAGCCCTTCAAATG	
474qRT *rfaH* RPv2	CCAGCGTTATTCATCAGCTTTC	
474qRT *rplD* FPv2	CCGCGGTACATCTTCTTGTTA	
474qRT *rplD* RPv2	TGCGCGTTCAGGTTCTATC	
474qRT *gyrA* FPv2	CTGGGTCTGGGAGTAGAGATTA	
474qRT *gyrA* RPv2	CGTGACGAATCCGACAAAGA	

### Validation of complementation of *dam* gene using qPCR

Bacterial cultures ST4/74, STΔ*dam*, and STΔ*dam* pBAD*^dam-np^* were initially grown until mid-logarithmic phase in Lennox Broth for the RNA isolation. One mL of the culture was centrifuged at 10,000 rpm for 5 minutes (Eppendorf, Germany). The pellet was resuspended in RNA stabilization solution (RNA Shield, Zymo Research, Irvine, CA, USA) and incubated on ice for 1 hour. RNA isolation was then performed using the DirectZol RNA isolation kit (Zymo Research, Irvine, CA, USA) following the manufacturer's protocol. The concentration and purity (260/280) of RNA were assessed using a NanoDrop One spectrophotometer (Thermo Fisher Scientific, USA). RNA from all cultures was normalized to 100 ng/μL prior to cDNA preparation. The reverse transcription was carried out using the commercial cDNA synthesis Kit (iScript cDNA synthesis kit, Bio-Rad Laboratories, Hercules, CA, USA) following the manufacturer's protocol, and the product was stored at −80°C. Quantitative real-time PCR was conducted on CFX Opus 96 Real-Time PCR System (Bio-Rad Laboratories, Hercules, CA, USA) with SsoAdvanced Universal SYBR Green Supermix (Bio-Rad Laboratories, Hercules, CA, USA). All cDNA samples were diluted to 1.25 ng/μL prior to the reaction. Each 10 μL reaction consists of 5 μL of master mix, 0.5 μL each of forward and reverse primer (10 μM stock), and 4 μL of diluted sample cDNA. Each PCR was performed with duplicate samples, and the cycling conditions were 2 min at 50°C, 2 min at 95°C, followed by 15 s at 95°C, 30 s at 58°C, and 30 s at 72°C for 40 cycles. In addition, a melt curve analysis was performed after amplification to verify product specificity. Relative quantification of the *dam* gene (ΔCt) was determined by normalization to the average value of three housekeeping genes (*rfaH, rplD,* and *gyrA*). PCR primers used in this study are described in Table 1. The relative abundance of the *dam* gene was calculated by using the 2^−ΔΔCt^ method.

### Construction of the *S*. enteritidis gene deletion mutants

The *S.* Enteritidis P125109 (SEN) mutants, SENΔ*hilD* and SENΔ*dam* were created in this study by deleting the respective genes in frame from the SEN chromosome using the λ Red recombinase one-step deletion protocol (Datsenko and Wanner, 2000). The oxidative stress mutants associated with both ST4/74 and SEN were also constructed in this study. Briefly, the kanamycin resistance cassette, along with the FRT (Flippase Recognition Target) recombination region, was amplified by PCR from plasmid pKD4 using pairs of 70-nucleotide primers that included 20 nucleotides targeting pKD4 as template DNA (Table 2). Homology extensions of 50 bp included 18 nucleotides of the 5ʹ end and 36 nucleotides of the 3ʹ end of the target gene. The PCR products were purified using the QIAquick PCR Purification Kit (QIAGEN, Hilden, Germany) and electroporated into electrocompetent SEN cells harboring recombinase-encoding plasmid pKD46. Allelic replacement of the kanamycin resistance gene cassette was confirmed by PCR, and nonpolar deletion mutants were obtained following the transformation of pCP20.

### Treatment replications and statistical interpretation

All experiments were carried out in triplicate. For the egg yolk and milk experiments, one 50 mL Falcon tube inoculated with either the wild type, mutant, or complementation strain was considered one experiment and was independently repeated thrice. For the meat experiments, meat pieces were drop-inoculated with the wild type, mutant, or complementation strain, with one piece per time point. The overall experiment was repeated 3 times to obtain triplicate data. The pairwise growth data of each mutant or the complementation strain grown in different food matrices were compared for statistical significance with those of the wild type using the Student’s *t-*test (*p* < 0.05). To perform a robust statistical analysis that accounts for repeated measurements over time, growth kinetics were analyzed using a linear mixed-effects model followed by post-hoc pairwise comparisons with p-value adjustments. All statistical analyses were performed in R v4.1.2..

## Results

### Growth kinetics of the ST4/74 regulatory protein mutants in egg yolk

First, as a control, we analyzed the proliferation of artificially inoculated ST4/74 in pasteurized egg yolk. The growth kinetics of ST4/74 in egg yolk revealed a lag phase of 4 hours, a log phase from 4 to 8 hours, and a stationary phase thereafter (Fig. 1). The proliferation of six of the eight tested transcriptional factor mutants (ST4/74Δ*ssrA,* ST4/74Δ*ssrB,* ST4/74Δ*rpoE,* ST4/74Δ*phoPQ,* ST4/74Δ*barA/sirA,* and ST4/74Δ*ompR*) did not exhibit any statistically significant difference compared to that of the wild type ST4/74 (Fig. 1). ST4/74Δ*hilD,* on the other hand, significantly grew better than the wild type (*p*-value 0.01871) (Fig. 2A). This latter observation is particularly significant because HilD is a crucial regulatory protein that regulates the expression of *Salmonella* Pathogenicity Island 1 (SPI-1) (Ellermeier and Slauch, 2007).

**Fig. 1:**
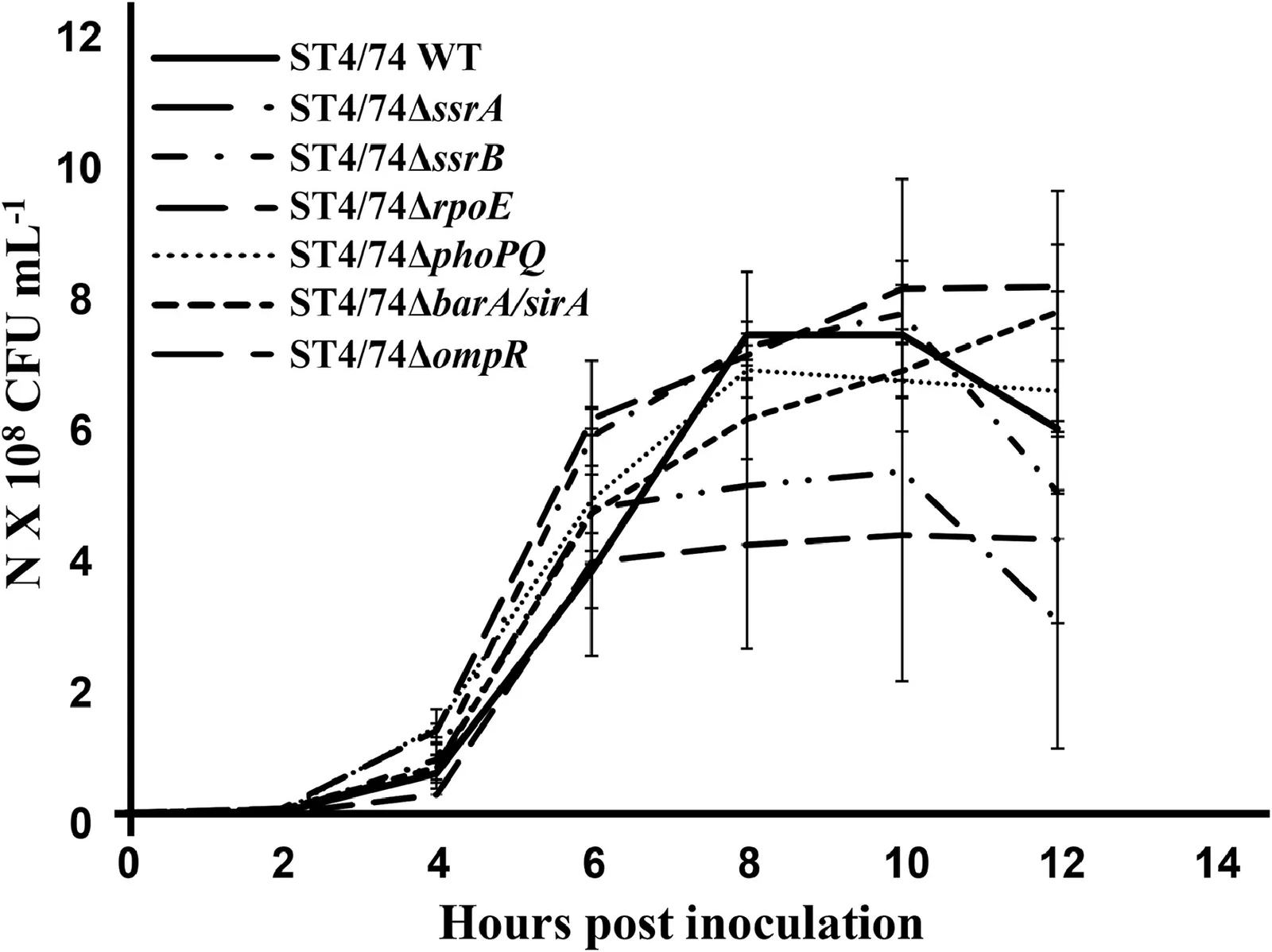
The bacterial growth curve of ST4/74 and six regulatory mutants showing no statistically significant difference from the wild type during proliferation in pasteurized liquid egg yolk. The graphs are based on cell counts averaged from three independent replicates.

**Fig. 2:**
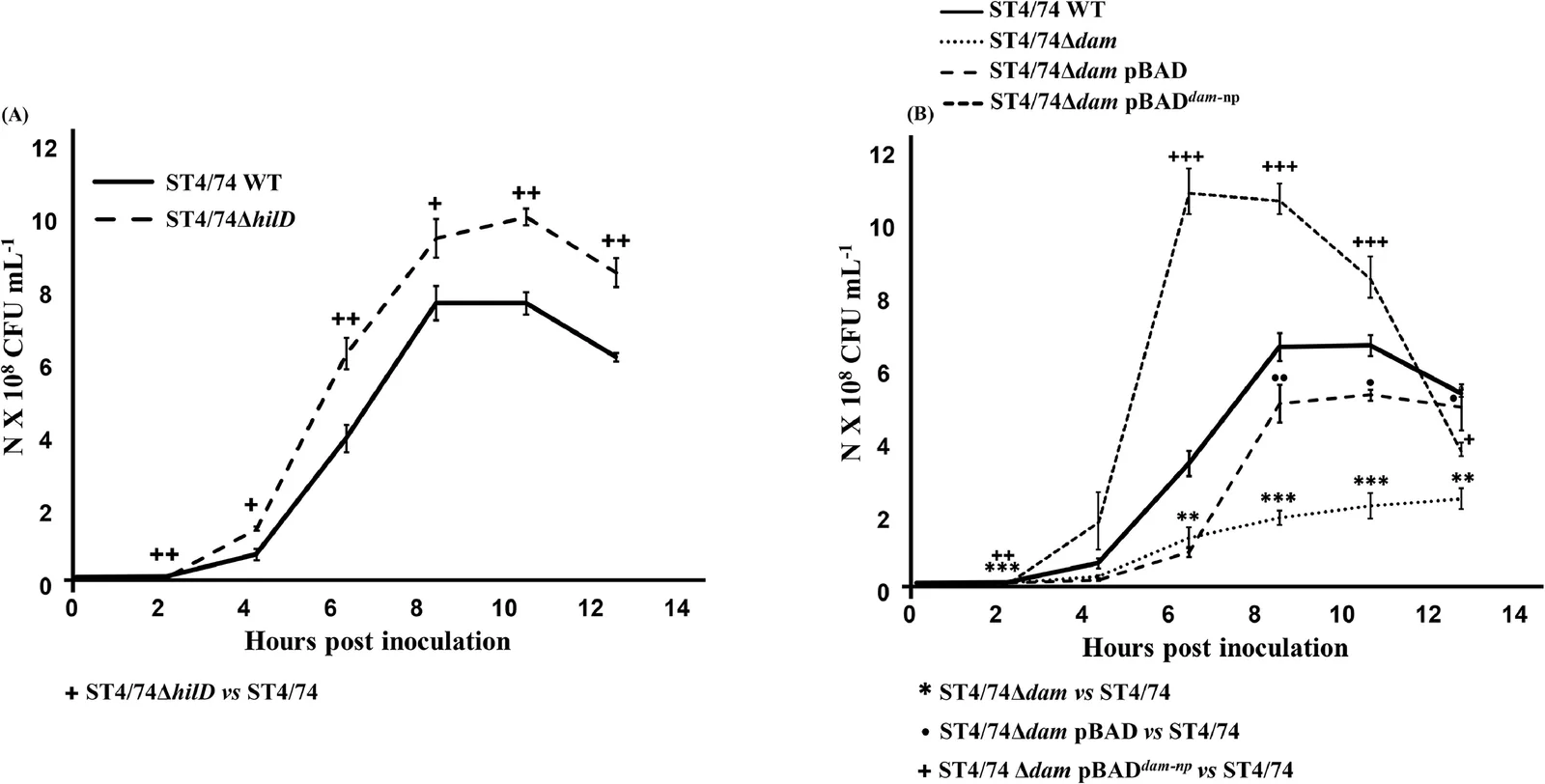
Bacterial growth curves of regulatory protein mutants showing altered growth phenotypes compared to the wild type ST4/74 in egg yolk. **(A)**. Growth curve of ST4/74Δ*hilD* showing hyper replication compared to the wild type. **(B)**. Growth curves of ST4/74Δ*dam*, ST4/74Δ*dam*pBAD, ST4/74Δ*dam* pBAD*^dam-np^*, showing the respective attenuation and complemented phenotypes compared to the wild type. Symbols indicate statistical significance of the pairwise comparison of each mutated strain to that of the wild type ST4/74 analyzed using Student’s t-*test p* = 0.05-0.01 (*)*, p* = 0.01–0.005 (**), and *p* < 0.005 (***).

### DNA adenine methyltransferase encoded by the gene *dam* is important for the proliferation of ST4/74 in egg yolk

Of the eight tested mutants, the deletion mutant of the *dam* gene encoding a DNA adenine methyltransferase, ST4/74Δ*dam*, showed a statistically significant 2- to 4-fold reduction (across all time points) in egg yolk proliferation compared to the wild type (*p-*value 0.002935) (Fig. 2B). The presence of the *dam* gene is, therefore, critical for *S*. Typhimurium survival in egg yolk. To further confirm the specificity of *dam* in egg yolk proliferation, we reintroduced the *dam* gene in ST4/74Δ*dam* by cloning the *dam* gene along with its native promoter into a pBAD plasmid (pBAD*^dam-np^*) and transforming pBAD*^dam-np^* into ST4/74Δ*dam,* generating ST4/74Δ*dam* pBAD*^dam-np^.* Here, the *dam* gene, along with the 300 bp upstream region containing its native promoter, replaced the l-arabinose-inducible P*ara* promoter on the pBAD plasmid. Since the *dam* gene is under the control of its native promoter, the expression of the *dam* gene will follow the native expression pattern during yolk survival. To first validate whether the complementation strain expresses the *dam* gene, we assessed the *dam* expression using RT-qPCR in the wild-type, Δ*dam* mutant, and ST4/74*Δdam* pBAD*^dam^*^-np^ during growth in LB. To ensure accurate quantification and avoid single-reference bias, transcript abundance was normalized against the geometric mean of three internal control genes (*rfaH, rplD,* and *gyrA*). The complemented strain exhibited *dam* expression, confirming significant restoration (*p-*value 0.0018) of *dam* transcription following complementation. The high expression of the *dam* gene in the complementation strain could be due to the high plasmid copy number of pBAD. To finally validate whether the *dam* complementation rescued the wild-type phenotype in Δ*dam,* ST4/74Δ*dam* pBAD*^dam-np^* was inoculated into egg yolk and grown for 14 hours. ST4/74Δ*dam* pBAD, carrying an empty pBAD vector, also functioned as an additional control to check whether the empty plasmid induced any effect in the egg yolk proliferation. The growth of ST4/74Δ*dam* pBAD did not differ significantly from that of ST4/74Δ*dam*, except at later timepoints (Fig. 2B)*.* However, the presence of ST4/74Δ*dam* pBAD*^dam-np^* significantly improved the growth of ST4/74Δ*dam* in egg yolk, confirming the specific effect of the *dam* gene in the proliferation of ST4/74 in egg yolk. The gene expression data, together with the restoration of the growth phenotype observed in the complemented strain, support the conclusion that the Δ*dam* phenotype was specifically attributable to the loss of *dam* function (Fig. 3).

**Fig. 3:**
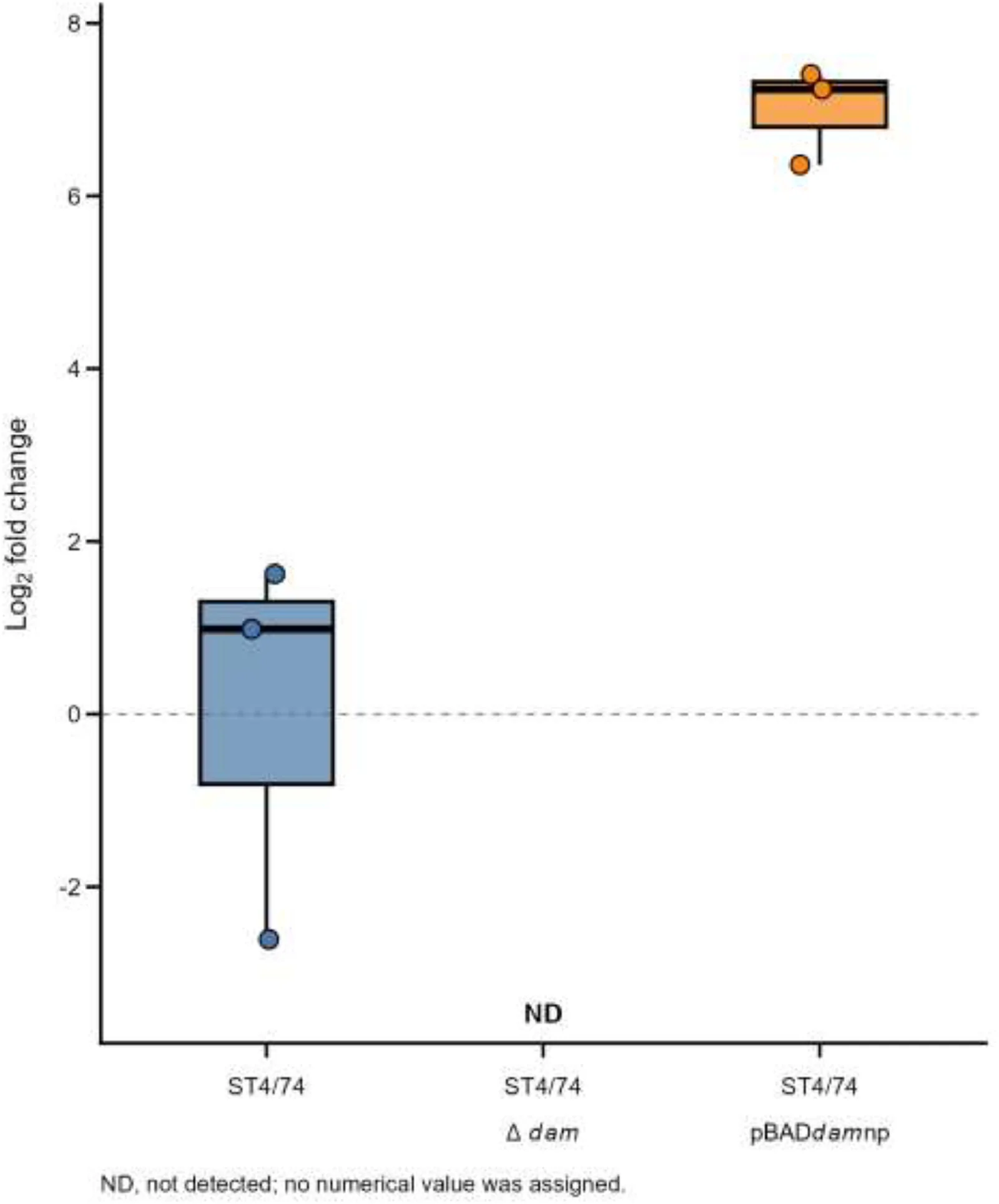
Expression levels of *dam* gene across *Salmonella enterica* serovar Typhimurium ST4/74 wild type, mutant and complemented strains. Data are presented as $\text{Lo}g_{2}$ fold change relative to ST4/74 wild type. Boxplots illustrate the median (horizontal line within the box), 25th and 75th percentiles (box boundaries), and minimum to maximum values (whiskers), with individual data points displayed (n=3). Groups evaluated include wild type ST4/74, the deletion mutant (Δdam), and the complemented strain (pBAD*^dam^*^-np^). ND = not detected (no numerical value was assigned).

### The presence of *dam* is critical for the ST4/74 proliferation in bovine milk and chicken meat

It was interesting to assess whether the presence of the *dam* gene was critical to the proliferation of ST4/74 in other food matrices relevant to *Salmonella* contamination, such as chicken meat and bovine milk. To test this, we inoculated ST4/74Δ*dam* into bovine milk & chicken meat and analyzed the growth kinetics over time. In bovine milk, ST4/74Δ*dam* showed significantly reduced growth compared to ST4/74 (*p-*value 0.007151), especially after 8 hours (Fig. 4A). The growth of the ST4/74Δ*dam* pBAD*^dam-np^*, on the other hand, was similar to ST4/74, showing that the *dam*-encoded DNA Adenine Methylase is critical for the survival of *S.* Typhimurium in milk. However, the growth of ST4/74Δ*dam* did not differ significantly from that of ST4/74 in chicken meat, although a slight inhibition was observed (Fig. 4B). This shows that the presence of *dam* could be dispensable for the survival of *S.* Typhimurium in chicken meat.

**Fig. 4:**
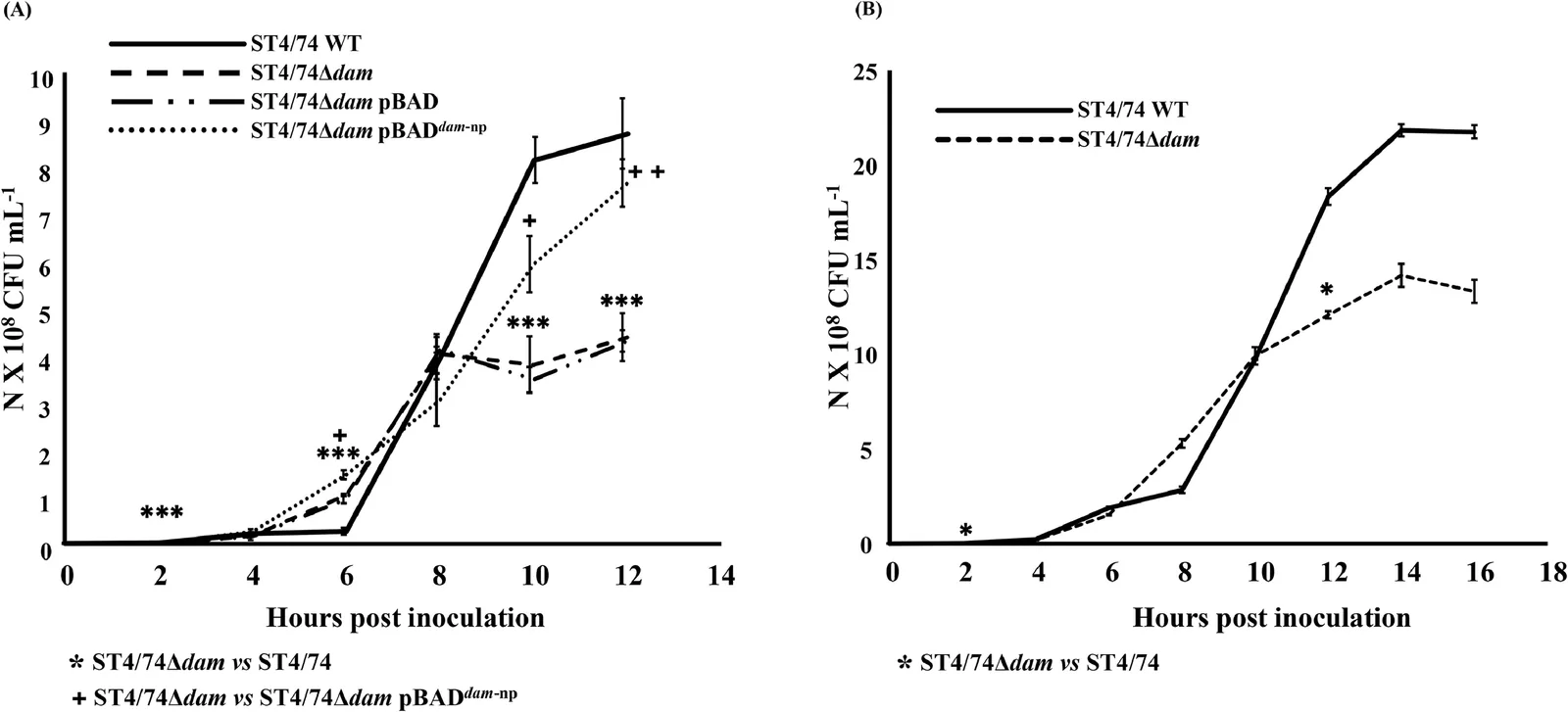
Growth curves of ST4/74Δ*dam*, ST4/74Δ*dam*pBAD, and ST4/74Δ*dam* pBAD*^dam-np^* in bovine milk **(A)** showing the respective attenuation and complemented phenotypes compared to the wild type, and chicken meat **(B)** showing no significant difference to the wild type. Symbols indicate statistical significance of the pairwise comparison of each mutated strain to that of the wild type ST4/74 analyzed using Student’s t-test *p* = 0.05-0.01 (*)*, p* = 0.01–0.005 (**), and *p* < 0.005 (***).

### S. enteritidis also requires *dam* and *hilD* for survival in egg yolk

It was interesting to evaluate whether the absence of *dam* and *hilD* affected egg yolk survival across different serotypes. For this, we first evaluated the growth kinetics of *S*. Enteritidis P125109 (SEN) in egg yolk. SEN entered the logarithmic phase at approximately 4 hours post inoculation, and the stationary phase at 10 hours post inoculation (Fig. 5). As in ST4/74, SENΔ*dam* showed reduced growth compared to the wild type SEN (*p-*value 0.0002209), while SENΔ*hilD* had improved growth (*p-*value 0.001026). Since the responses of both transcriptional factor mutants are comparable between *S.* Typhimurium and *S.* Enteritidis, we anticipate that both transcriptional factors play consistent roles in the proliferation of *Salmonella* in egg yolk.

**Fig. 5:**
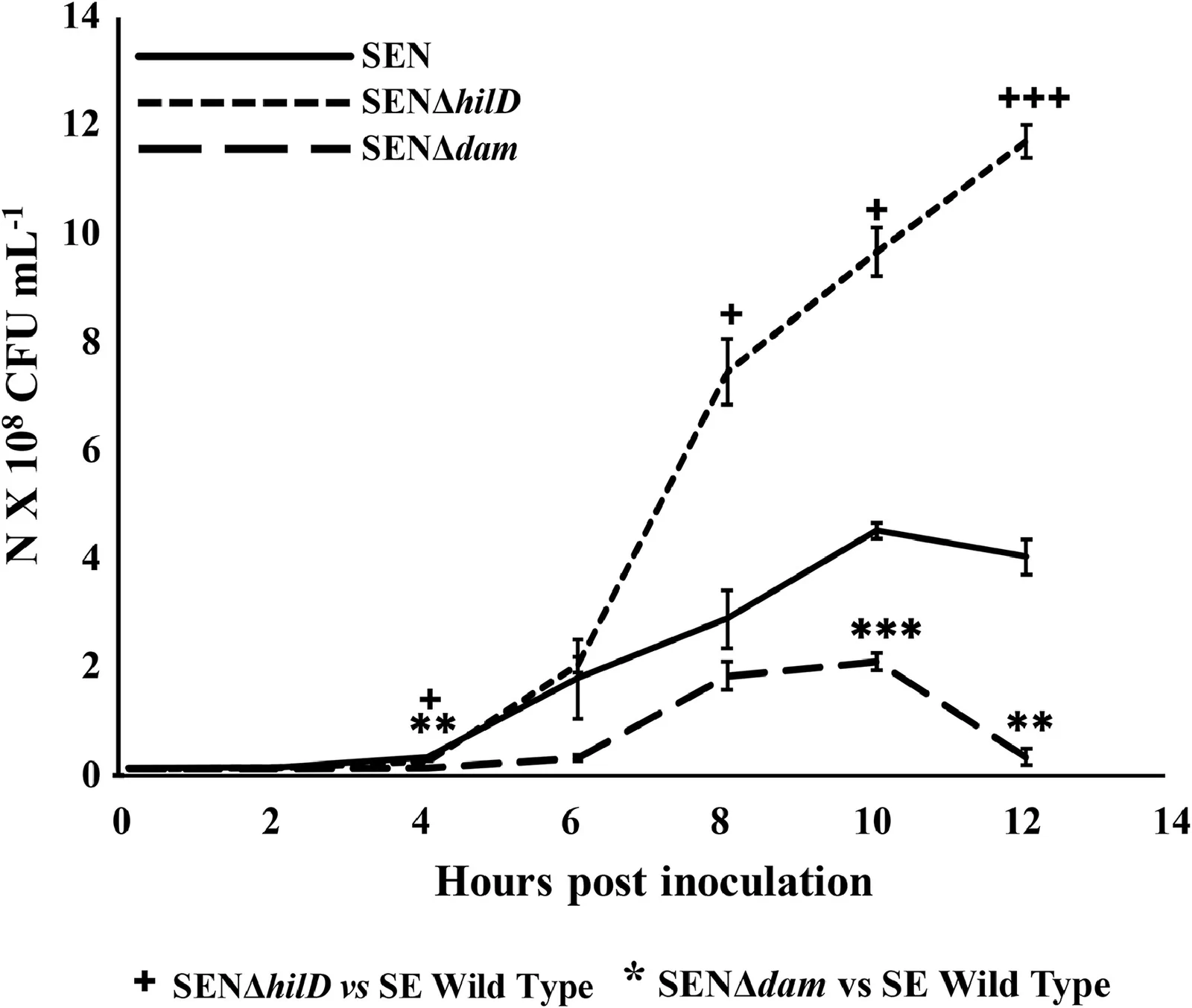
Growth kinetics depicting altered phenotypes of SENΔ*hilD* and SENΔ*dam* compared to wild type SEN during proliferation in pasteurized egg yolk. Symbols indicate statistical significance of the pairwise comparison of each mutated strain to that of the wild type SEN analyzed using Student’s t-test *p* = 0.05-0.01 (*)*, p* = 0.01–0.005 (**), and *p* < 0.005 (***).

### Phenotypic evidence for the involvement of oxidative stress response genes in egg yolk survival

Next, we determined whether the oxidative stress response contributed to *Salmonella* egg yolk proliferation. To test this, we created a deletion mutant of *soxS,* a transcriptional factor that modulates *Salmonella*'s survival to oxidative stress (Fang, Vazquez-Torres and Xu, 1997). In egg yolk, ST4/74Δ*soxS* had an extended lag phase of 8 hours. However, at later time points, ST4/74Δ*soxS* exhibited significant overgrowth (p-value = 0.00001807) compared to the wild type, particularly at 14 and 16 hours (Fig. 6A). To further confirm the importance of the oxidative stress response, we generated deletion mutants of the genes encoding catalase (*katN*), superoxide dismutase (*sodA*), and an Mn^2+^ transporter (*mntH*), all of which are known to play critical roles in *Salmonella*'s oxidative stress response. In egg yolk, ST4/74Δ*katN,* ST4/74Δ*sodA*, and ST4/74Δ*mntH* all exhibited an increased lag phase (6 hours) compared to the wild type (4 hours). However, beyond the lag phase, all three mutants significantly improved their growth compared to the wild type (*P* value 0.00007987, < 0.001, 0.0002375, for ST4/74Δ*katN,* ST4/74Δ*sodA*, and ST4/74Δ*mntH,* respectively) (Fig. 6A). For the oxidative stress response mutants, too, the phenotypic effects were consistent across serotypes. The oxidative stress response mutants of SEN, *viz.* SENΔ*katN,* SENΔ*sodA*, and SENΔ*mntH* also showed reduced growth until 6 hours post-inoculation, beyond which increased growth was noted compared to that of the wild type (Fig. 6B).

**Fig. 6:**
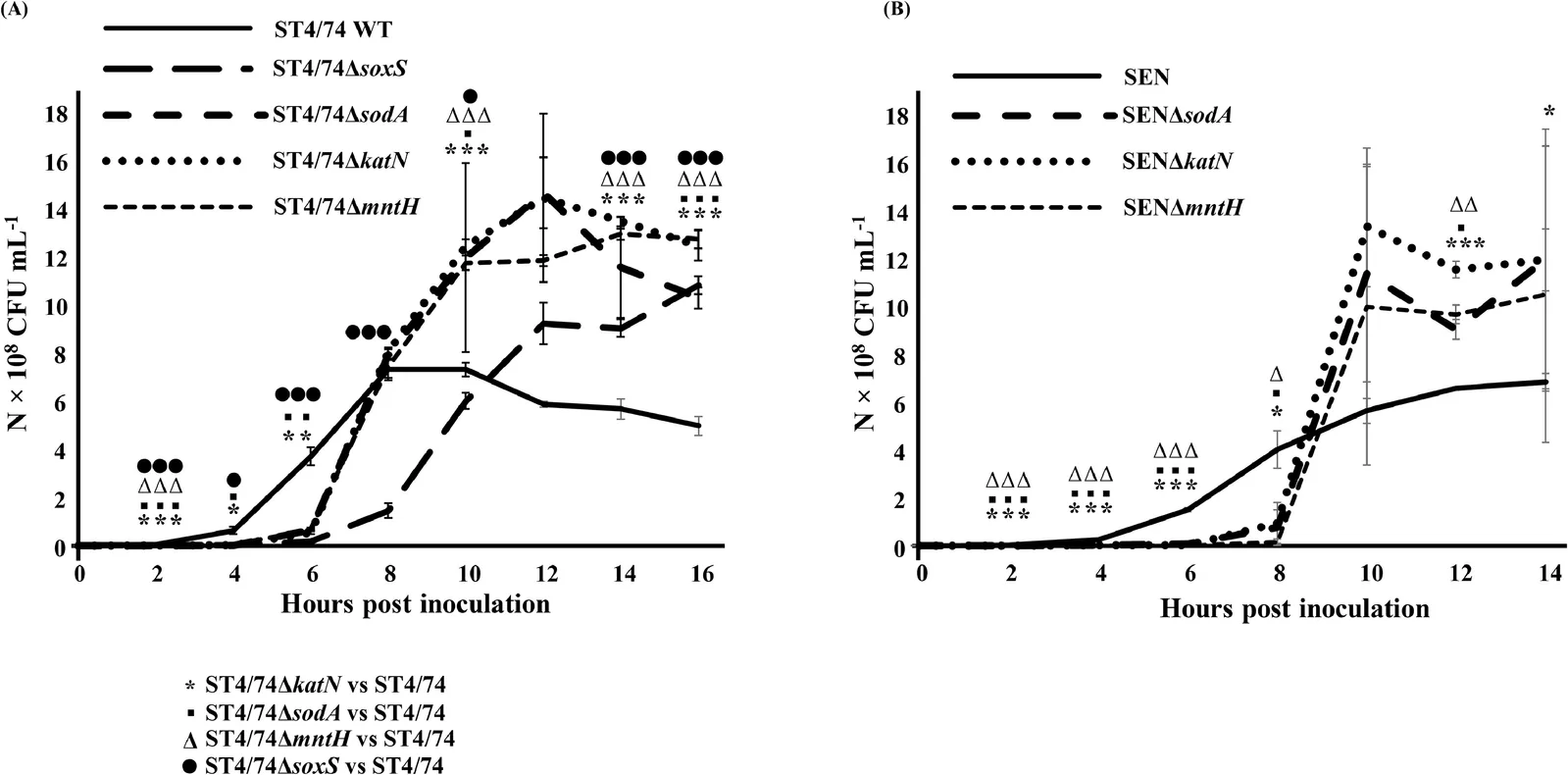
Growth kinetics of the oxidative stress mutants of both ST4/74 **(A)** and SEN **(B)** during proliferation in pasteurized egg yolk. Symbols indicate statistical significance of the pairwise comparison of each mutated strain to that of the wild type ST4/74 or SEN analyzed using Student’s t-test *p* = 0.05-0.01 (*)*, p* = 0.01–0.005 (**), and *p* < 0.005 (***).

## Discussion

Our primary goal was to characterize the physiology of *S.* Typhimurium during proliferation in egg yolk. We used *S.* Typhimurium strain ST4/74 (ST4/74) as a model organism for our studies primarily due to two reasons: (1) the genome and infection-associated transcriptomes of ST4/74 are well characterized (Kroger, Colgan, Srikumar, Handler, Sivasankaran, Hammarlof, Canals, Grissom, Conway, Hokamp and Hinton, 2013; Srikumar, et al., 2015), and (2) the regulon of different infection-associated transcriptional factors is well characterized (Colgan, Kroger, Diard, Hardt, Puente, Sivasankaran, Hokamp and Hinton, 2016). Studies involving DNA-binding transcriptional factors played a major role in elucidating the biology of *Salmonella* during infection (Fabrega and Vila, 2013). The stresses faced by bacteria during survival in food matrices and host pathogenesis are similar (Marmion, et al., 2022). Therefore, we hypothesized that well-characterized infection-associated transcriptional factors could be critical for *S*. Typhimurium proliferation in egg yolk. To test this, we employed a suite of well-characterized regulatory mutants (Colgan, Kroger, Diard, Hardt, Puente, Sivasankaran, Hokamp and Hinton, 2016) to physiologically profile the proliferation of *S.* Typhimurium in egg yolk.

The proliferation of six of the eight ST4/74 transcriptional factor mutants was similar to that of the wild type, showing no significant role in the egg yolk survival. However, *dam* (encoding DNA adenine methyltransferase) is critical for the proliferation of both *S.* Typhimurium ST4/74 and *S.* Enteritidis P125109 in pasteurized egg yolk. The enzyme methylates N-6 adenine of GAnTC, and this methylation has numerous consequences in bacterial physiology, including DNA segregation, mismatch repair, chromosome replication, and transcriptional response (Giacomodonato, et al., 2009). Structurally, egg yolk is composed of non-soluble protein aggregates (granules) suspended in a clear yellow plasma containing low-density lipoproteins and soluble proteins (Anton, 2013). Granules, on the other hand, represent 22% of the yolk dry matter, containing 50% of yolk proteins and 7% of lipids. Phosvitin, a major yolk protein, represents 16% of the granular content (Anton, 2013) and exhibits potent antimicrobial activity by binding strongly to metal ions, such as iron and calcium, thereby reducing their bioavailability to pathogens (Ishikawa, et al., 2007). This can disrupt bacterial cell membranes, causing leakage of cellular contents, ultimately leading to bacterial cell death (Choi, et al., 2004). Deletion of the *dam* gene results in an altered bacterial envelope with compromised membrane integrity and an unstable peptidoglycan structure (Pucciarelli, et al., 2002). Consequently, the *dam* null mutant could be more susceptible to membrane-targeting stressors such as Phosvitin in egg yolk, leading to pronounced growth attenuation in egg yolk. Because egg yolk contains high levels of membrane-disruptive components like phosvitin, it is possible that Dam-dependent envelope stability serves a protective role against phosvitin-mediated membrane stress, thereby allowing wild type *Salmonella* to proliferate in egg yolk. However, for various technical reasons, we did not carry out a direct phosvitin challenge in any of the mutants tested here. Therefore, the specific involvement of phosvitin and the role of Dam in *Salmonella’s* response to Phosvitin stress is hypothetical and requires further experimental validation.

Dam-mediated membrane stability is therefore critical for *Salmonella* egg yolk survival. In addition to egg yolk proliferation, null mutants of *dam* were severely attenuated for survival in bovine milk, confirming its critical role in *Salmonella* survival in multiple food matrices. The severe attenuation of the *dam* null mutants in multiple animals has led to the strain being proposed as a vaccine strain (Dueger, et al., 2001, 2003). However, being a transcriptional factor, Dam is also shown to regulate fimbrial gene expression, effector protein secretion, and inflammatory responses in macrophages in *Salmonella* (Giacomodonato, Sarnacki, Llana and Cerquetti, 2009). It is unclear whether any or all of the above *dam*-associated physiologies cumulatively contribute to *Salmonella* proliferation in egg yolk and bovine milk. Furthermore, the importance of the *dam*-encoded DNA adenine methyltransferase in both egg yolk and bovine milk suggests that bacterial pathogens face similar stresses during survival in various food matrices. More studies are needed to elucidate the distinct stress responses common to different bacterial pathogens during survival in various food matrices.

Interestingly, some of the tested mutants grew significantly better than the wild type during proliferation in egg yolk. A typical example is *hilD*, where mutants from both *S.* Typhimurium ST4/74 and *S.* Enteritidis P125109 overgrew the wild type. Similarly, null mutants of oxidative stress response genes such as *katN, mntH,* and *sodA* from both *S.* Typhimurium ST4/74 and *S.* Enteritidis P125109 also outgrew their respective wild types, particularly at later time points.

Of the 24 *Salmonella* pathogenicity islands (SPIs) identified, SPI-1 and SPI-2 are the most well-characterized (Sia, Pearson, Howden, Williamson and Ingle, 2025). Genes on the SPI-1 island encode a Type III secretion system that enables *Salmonella* to invade enterocytes, while genes on the SPI-2 island support intracellular survival (Sia, Pearson, Howden, Williamson and Ingle, 2025). HilD is an AraC-like transcriptional regulator that induces the expression of HilA (Schechter, Damrauer and Lee, 1999; Schechter and Lee, 2001). HilA is the master regulator of the SPI-1 island (Bajaj, Hwang and Lee, 1995). HilD, along with HilC and RtsA controls the *hilA* expression by a feed-forward loop, in turn, activating the SPI-1 T3SS machinery (Ellermeier, Ellermeier and Slauch, 2005). HilD also modulates the expression of *ssrAB*, which encodes the two-component system that acts as the central positive regulator of the SPI-2 genes (Bustamante, et al., 2008). Consequently, the *hilD* mutant is highly attenuated for invasion in a chicken model (Gomez-Chavez, et al., 2025) and vegetables (Arista-Regalado, et al., 2023). Since all the above conditions involve an invasion phenotype, and HilD is a positive regulator of invasion, the *hilD* mutant's invasion-deficient phenotype is understandable. Surprisingly, in egg yolk, the *hilD* mutant of both *S.* Typhimurium and *S.* Enteritidis outgrew the wild type. In non-host environments *in vitro*, only a subset of *Salmonella* cells expresses *hilA*-mediated type III secretion systems (Sturm, et al., 2011). These type III secretion-positive cells grow more slowly than the type III secretion system-negative cells, implying that expression of the secretion system imposes a growth penalty on the bacterial cells. Subsequently, *hilA* and *hilD* mutants outcompeted the wild type in mixed cultures (Sturm, Heinemann, Arnoldini, Benecke, Ackermann, Benz, Dormann and Hardt, 2011). The *hilD* gene in *S.* Enteritidis was up-regulated more than 10-fold during survival in egg yolk (Xu, et al., 2022). Therefore, upregulation of *hilD* could impose a growth penalty on *Salmonella* growth in egg yolk and milk. Deletion of *hilD* could alleviate this growth penalty, resulting in a hyper-replicative phenotype, as observed in our data. This observation, however, needs further experimental validation.

Similarly, mutants of both *S.* Typhimurium and *S.* Enteritidis that were incapable of eliciting a successful oxidative stress response also grew better than the wild type during proliferation in egg yolk, suggesting a potential role for oxidative stress response in egg yolk adaptation. Egg yolk is known to be rich in polyunsaturated fatty acids. Growth of *Salmonella* can induce lipid peroxidation in egg yolk (Afshordi, et al., 2016). Egg yolk is rich in iron (Bashir, et al., 2015) that can induce Fenton’s reaction in *Salmonella*. Both lipid peroxidation and the Fenton reaction expose *Salmonella* to oxidative stress, necessitating a robust oxidative stress response. Although the altered growth kinetics of the *Salmonella* oxidative stress response mutants are unsurprising, the mutants' improved growth compared to the wild type is intriguing. However, our observations are based solely on the growth kinetics of the oxidative stress response mutants. Further experiments involving antioxidants, ROS measurements, and expression of oxidative stress-related genes are needed to validate our observations.

In conclusion, we show that *dam, hilD,* and oxidative stress response genes play an extended role beyond host pathogenesis, and into food matrix survival. However, since most virulence-associated regulatory mutants did not exhibit restricted growth in egg yolk, we believe *Salmonella* yolk survival is modulated by a separate set of hitherto uncharacterized regulatory proteins whose expression is specific to food matrices. Overall, we provide early insights into the molecular mechanisms underlying *Salmonella* proliferation in egg yolk.

## Author Contributions

**Aisha Madi** – played a major role in the conceptualization, methodology, validation, formal analysis, investigation, and data curation. Aisha also played a major role in writing both the original draft and the revised versions. **Greeshma Bharathan** – also played a major role in conceptualization, methodology, validation, formal analysis, investigation, and data curation. Greeshma contributed substantially to writing both the original draft and the revised versions. **Ana Victoria Troncoso** – contributed to methodology, validation, formal analysis, investigation, and data curation. Ana contributed substantially to reviewing/editing the original and the revised versions of the draft. **Camila Ortiz Melcon** – also contributed to methodology, validation, formal analysis, investigation, and data curation. Camilla reviewed and edited the original/revised versions of the manuscript. **Karoll Rodriguez Chinchilla** – contributed to the methodology, validation, and data curation. Karoll also participated in writing the original draft and in review/editing. **Maddie Childress** – participated as an undergraduate research student in the methodology, validation, and data curation. Michelle also helped review both the original draft and the review/editing. **Michelle Hayden** – contributed to the methodology, formal analysis, investigation, and data curation. Michelle also helped in reviewing both the original draft and the revised versions. **Krishna Pada Singha** – played a major role in the methodology, validation, formal analysis, and data curation. Krishna also contributed to the review and editing of the revised version of the manuscript. **Amit Morey** – Contributed to the conceptualization, co-supervision, and co-project administration. Amit also contributed to the review and editing of the original and revised versions of the manuscript. **Stephen White** – played a major role in the funding acquisition, conceptualization, and project administration. Stephen also contributed to the review of both the original and revised versions of the manuscript. **Shabarinath Srikumar** – played the pivotal role in conceptualization, resource generation, funding acquisition, project administration, and student supervision. Srikumar also co-wrote the original version of the manuscript and played a major role in revising it in response to the reviewers' comments.

## Disclosures

The authors declare that there are no competing interests.
